# Supplementary material for: Alleviating oxygen evolution from Li-excess oxide materials through theory-guided surface protection
Source: Nat Commun. 2018 Nov 2;9:4597. doi: 10.1038/s41467-018-07080-6 (PMC6214920; doi:10.1038/s41467-018-07080-6)
Supplement: Supplementary file 1 — Supplementary Information [file 41467_2018_7080_MOESM1_ESM.pdf]

Alleviating Oxygen Evolution from Li-excess Oxide Materials through Theory-guided Surface Protection

Supplementary Information

Yongwoo Shin[1], Wang Hay Kan[1], Muratahan Aykol[1], Joseph K Papp[2], Bryan McCloskey[1,2], Guoying Chen[1], and Kristin A. Persson[1,3 *]

[1] Lawrence Berkeley National Laboratory, Berkeley, CA 94720, USA

[2] Department of Chemical and Biomolecular Engineering, University of California Berkeley, Berkeley, CA 94704, USA

[3] Department of Materials Science and Engineering, University of California Berkeley, Berkeley, CA 94704, USA

* kapersson@lbl.gov

Supplementary Note 1: Computations

Supplementary Table 1. Oxidation states and ionic radius for the dopants

Dopant	Oxidation	Ionic radius
Al	3	0.530
Si	4	0.400
Sc	3	0.745
Ti	4	0.605
V	4	0.580
Cr	3	0.615
Fe	4	0.585
Co	4	0.530
Ni	4	0.480
Cu	3	0.540
Zn	2	0.740
Ga	3	0.620
Ge	4	0.530
As	3	0.580
Y	3	0.900
Zr	4	0.720
Nb	5	0.640
Mo	5	0.650
Ru	4	0.620
Rh	3	0.665
Pd	4	0.615
Ag	3	0.750
Cd	2	0.950
In	3	0.800
Sn	4	0.690
Sb	5	0.600
Hf	4	0.710
Ta	5	0.640
W	6	0.600
Re	4	0.630
Os	4	0.630
Ir	4	0.625
Pt	4	0.625
Au	3	0.850
Hg	2	1.020
Tl	3	0.885
Pb	4	0.775
Bi	5	0.760

Supplementary Table 2. Stable Polymorphs of each dopant

Dopant	Composition 1	Composition 2	Composition 3	Composition 4
Al	LiAlO2	Li2MnO3	Li2O2	
Si	Li2MnO3	Li2SiO3		
Sc	Li2MnO3	Li2O2	LiScO2	
Ti	Li2MnO3	Li2TiO3		
V	Li2MnO3	LiMnO2	Li3VO4	
Cr	Li2MnO3	Li3CrO4	LiCrO2	
Fe	Li2FeO3	Li2MnO3		
Co	Li7Co5O12	Li2O2	Li2MnO3	
Ni	Li2NiO3	Li2MnO3		
Cu	LiCuO2	O2	Li3CuO3	Li2MnO3
Zn	Li2O2	Li2MnO3	ZnO	
Ga	LiGaO2	Li2O2	Li2MnO3	
Ge	Li2GeO3	Li2MnO3		
As	Li3AsO4	LiMnO2	Li2MnO3	
Y	Li2O2	LiYO2	Li2MnO3	
Zr	Li2MnO3	Li2ZrO3		
Nb	Li3NbO4	LiMnO2	Li2MnO3	
Mo	Li2MnO3	LiMnO2	Li4MoO5	
Ru	Li2MnO3	Li2RuO3		
Rh	Li2RhO3	Li2MnO3		
Pd	Li2MnO3	Li2PdO3		
Ag	Li2O2	LiAgO2	Li2MnO3	
Cd	Li2O2	CdO	Li2MnO3	
In	LiInO2	Li2MnO3	Li2O2	
Sn	Li2MnO3	Li2SnO3		
Sb	Li2MnO3	LiMnO2	Li3SbO4	
Hf	Li2HfO3	Li2MnO3		
Ta	LiMnO2	Li3TaO4	Li2MnO3	
W	Li2MnO3	LiMnO2	Li4WO5	
Re	LiMnO2	Li5ReO6	Li2MnO3	
Os	Li7OsO6	Os	OsO2	Li2MnO3
Ir	Li2IrO3	Li2MnO3		
Pt	Li2PtO3	Li2MnO3		
Au	Li3AuO3	O2	LiAuO2	Li2MnO3
Hg	Li2MnO3	Li2O2	HgO	
Tl	Tl2O3	Li5TlO4	Li2MnO3	Li2O2
Pb	Li2MnO3	Li2PbO3		
Bi	Li2MnO3	Li3BiO4	LiBiO2	

For example, 25% of Al surface doping presented in following chemical equation on the MaterialsProject database;

$$E^{eq}_{PD}(\text{Al on (001)}) = 0.014*E(\text{LiAlO2}) + 0.028*E(\text{Li2MnO3}) + 0.95*E(\text{Li2O2})$$

Supplementary Note 2: Experiments

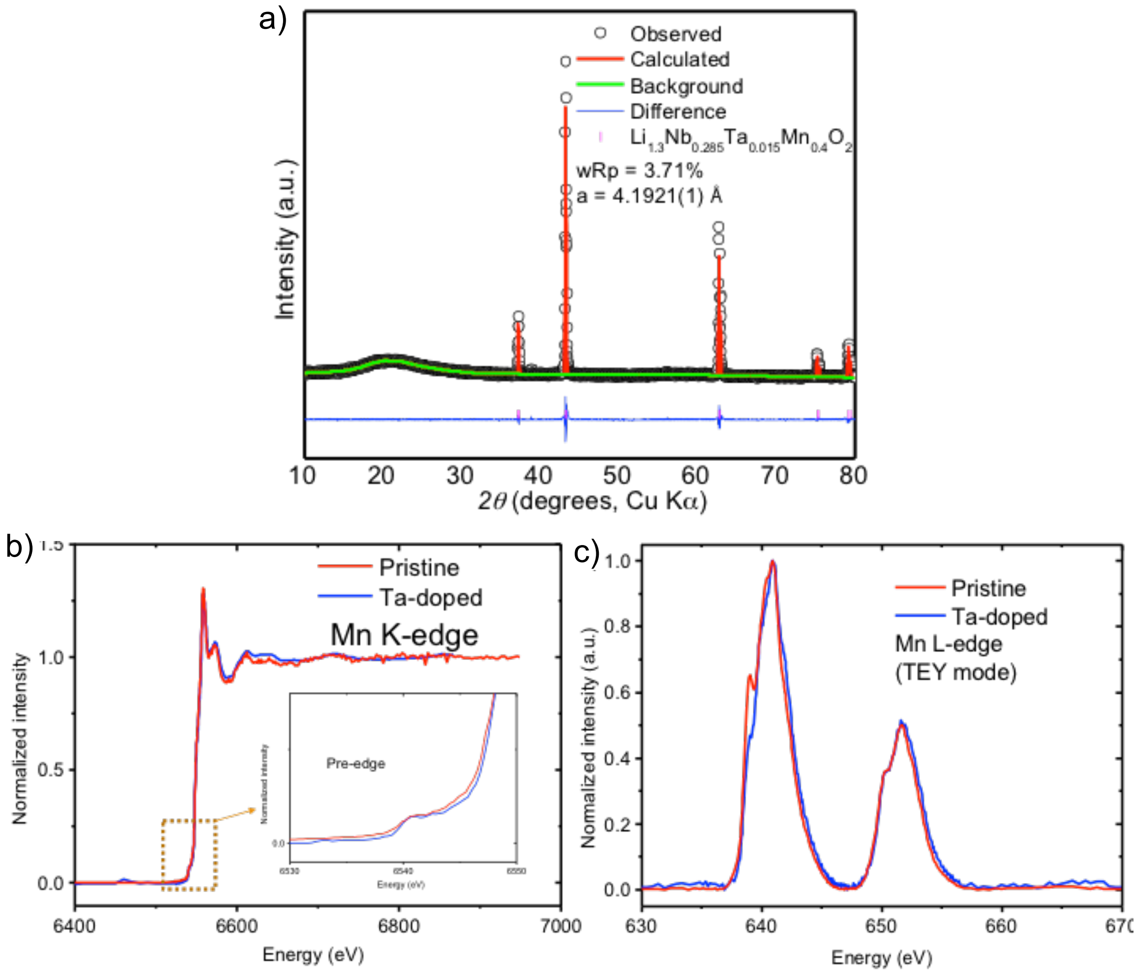

Supplementary Figure 1. Rietveld refinement of synchrotron XRD pattern collected on as-prepared $Li_{1.3}Nb_{0.285}Ta_{0.015}Mn_{0.4}O_2$ (a). Hard (b) and soft (c) X-ray absorption spectra (XAS) of the as-prepared $Li_{1.3}Nb_{0.285}Ta_{0.015}Mn_{0.4}O_2$ and $Li_{1.3}Nb_{0.3}Mn_{0.4}O_2$.

PXRD Rietveld refinement indicates that the crystal structure of the as-prepared $Li_{1.3}Nb_{0.285}Ta_{0.015}Mn_{0.4}O_2$ and pristine $Li_{1.3}Nb_{0.3}Mn_{0.4}O_2$ are identical, and both have cell parameter of ca. 4.195 Å with a space group of *Fm-3m* (225). Hard (b) and soft (c) XAS were used to understand the impact of the Ta-dopant on the Mn valence state in lithium-rich samples. As shown in Supplementary Figure 1-(b) the Mn K-edge in the hard XAS spectra, the absorption edge energy, which was determined at the intensity equal to 0.5 of the normalized spectra, was 6548.9 eV for both samples. This indicates that the bulk Mn valence state was un-altered upon doping. In addition, there was no change in the pre-edge region

(Supplementary Figure 1-(b) inlet) suggesting that the coordination environment for the Mn ions was identical for both samples. For the surface valence state characterization, the Mn L-edge in the soft XAS in total electron yield (TEY; up to 5 nm) was investigated. The spectra were found to be overlapped with each other with L_{III} and L_{II} centered at 640.9 and 651.7 eV, respectively. Both results suggested that the Ta dopant did not alter the bulk and surface valence state of Mn ions.

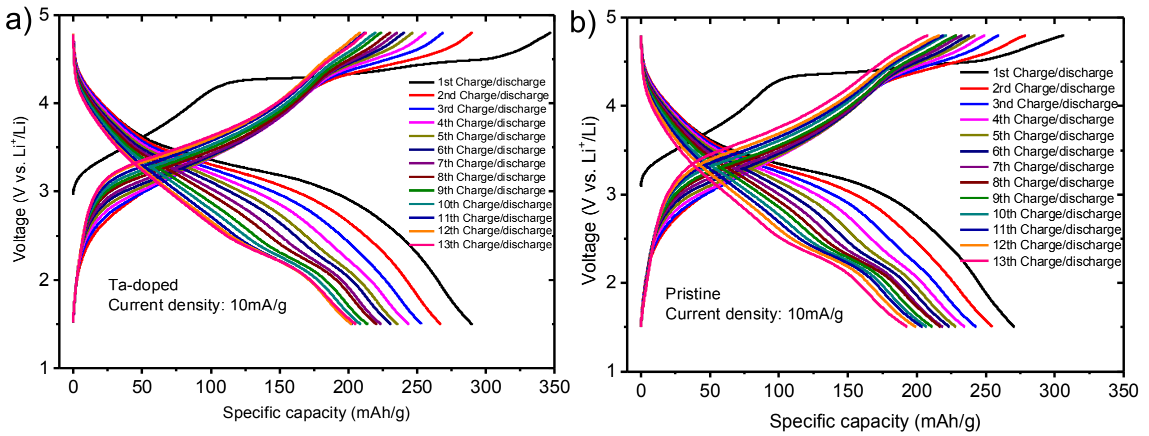

Supplementary Figure 2. The charge and discharge profiles of Ta-doped $Li_{1.3}Nb_{0.285}Ta_{0.015}Mn_{0.4}O_2$ (a) and the pristine $Li_{1.3}Nb_{0.3}Mn_{0.4}O_2$ (b).

The electrochemical performance was evaluated in both galvanostatic and cyclic voltammetric modes. For the galvanostatic mode, both cells were cycled at 10 mA/g between 1.5 and 4.8 V. As shown in Supplementary Figure 2, the Ta-doped sample had slight higher specific capacity for both charging and discharging cycle, mainly due to the lower polarization which might relate to the mitigation of the unwanted surface reaction at high voltage regime. As the cells were cycled, voltage fading and capacity reduction were observed. After 10 cycles, the specific capacity of the Ta-doped sample was higher than that of the pristine for both charging and discharging cycles. The cyclic voltammograms (CV) were also performed to see if there was a change of the reaction mechanism which might show additional peaks at different voltages. The CV of both samples were very similar between the pristine and the Ta-doped samples, suggested that there was no alternation of the bulk reaction mechanism for Mn ions and/or O ions upon doping.

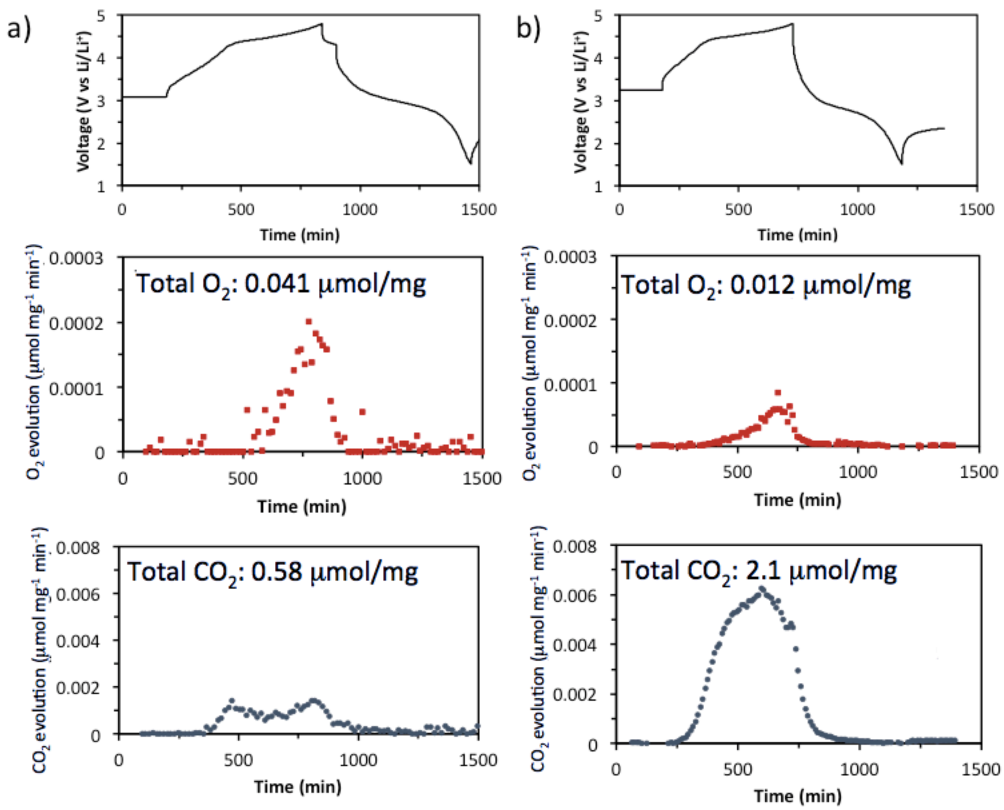

Supplementary Figure 3. Differential electrochemical mass spectroscopy (DEMS) of the pristine $Li_{1.3}Nb_{0.3}Mn_{0.4}O_2$ (a) and Ta-doped $Li_{1.3}Nb_{0.285}Ta_{0.015}Mn_{0.4}O_2$ (b). Gas evolution rates are normalized by weight of active material. The pristine material includes an OCV interval between charge and discharge, which were both performed at C/10 rates.

Differential electrochemical mass spectrometry (DEMS) was performed to monitor gas evolution from pristine and Ta-doped LNMO. The DEMS experimental setup is described completely elsewhere.[1,2] Cells were prepared by sandwiching a 10 mm diameter Li metal foil, a QMA Whatman glass fiber separator (dried at 200°C prior to use) with 1M LiPF6 in 1:1 EC/DMC as the electrolyte (100 μL), and a 12 mm diameter cathode in a custom-built hermetically sealed cell. Cathodes were prepared by mixing active material, carbon black, and polyvinylidene fluoride binder at a ratio of 8:1:1. N-methyl-2-pyrollidone was used to make a slurry with this mixture, which was then spread on stainless steel mesh with a typical loading of 10mg per cathode. Cathodes were dried at 200°C overnight prior to use. CO_2 evolution is correlated with residual lithium carbonate used in synthesis of the active material.[2] Simple titrations of the pristine active materials indicated that the $Li_{1.3}Nb_{0.3}Mn_{0.4}O_2$ contained 0.85wt% Li_2CO_3, and the Ta-doped $Li_{1.3}Nb_{0.285}Ta_{0.015}Mn_{0.4}O_2$ contained 5.74wt% Li_2CO_3. This increase can be attributed to the excess Li_2CO_3 used for the synthesis of the Ta-doped material.

Supplementary References

1. McCloskey, B. D., Bethune, D. S., Shelby, R. M., Girishkumar, G. & Luntz, A. C. Solvents' Critical Role in Nonaqueous Lithium–Oxygen Battery Electrochemistry. *J. Phys. Chem. Lett.* **2,** 1161–1166 (2011).
2. Renfrew, S. E. & McCloskey, B. D. Residual Lithium Carbonate Predominantly Accounts for First Cycle CO2 and CO Outgassing of Li-Stoichiometric and Li-Rich Layered Transition-Metal Oxides. *J. Am. Chem. Soc.* **139,** 17853–17860 (2017).
